# Persistence and compliance to antidepressant treatment in patients with depression: A chart review

**DOI:** 10.1186/1471-244X-9-38

**Published:** 2009-06-16

**Authors:** Norifusa Sawada, Hiroyuki Uchida, Takefumi Suzuki, Koichiro Watanabe, Toshiaki Kikuchi, Takashi Handa, Haruo Kashima

**Affiliations:** 1 Department of Psychiatry, Saiseikai Central Hospital 1-4-17 Mita, Minato-ku, Tokyo, 108-0073, Japan; 2 Department of Neuropsychiatry, Keio University, School of Medicine35 Shinanomachi, Shinjuku-ku, Tokyo, 160-8582, Japan; 3 Department of Psychiatry, University of Toronto250 College Street, Toronto, Ontario, M5T 1R8, Canada; 4 Geriatric Mental Health Program, Centre for Addiction and Mental Health1001 Queen Street West, Toronto, Ontario, M6J 1H4, Canada; 5 Department of Psychiatry, Inokashira Hospital 4-14-1 Kamirenjaku, Mitaka-shi, Tokyo, 181-8531, Japan

## Abstract

**Background:**

Adherence has recently been suggested to be divided into these two components: persistence (i.e., whether patients continue treatment or not) and compliance (i.e., whether patients take doses as instructed). However, no study has yet assessed these two clinically relevant components at the same time in adherence to antidepressant treatment in the clinical outpatient setting.

**Methods:**

In this retrospective chart-review, 6-month adherence to antidepressants was examined in 367 outpatients with a major depressive disorder (ICD-10) (170 males; mean ± SD age 37.6 ± 13.9 years), who started antidepressant treatment from April 2006 through March 2007. Additionally, we evaluated Medication Possession Rate (MPR), defined as the total days a medication was dispensed to patients divided by the treatment period.

**Results:**

Only 161 patients (44.3%) continued antidepressant treatment for 6 months. Among 252 patients who discontinued their initial antidepressant, 63.1% of these patients did so without consulting their physicians. Sertraline use was associated with a higher persistence rate at month 6 (odds ratio 2.59 in comparison with sulpiride), and the use of anxiolytic benzodiazepines had a positive effect on persistence to antidepressant treatment only at month 1 (odds ratio 2.14). An overall MPR was 0.77; 55.6% of patients were considered compliant (i.e., a MPR of ≥ 0.8).

**Conclusion:**

Given a high rate of antidepressant discontinuation without consulting their physicians, closer communication between patients and their physicians should be encouraged. Although the use of anxiolytic benzodiazepines was associated with a higher persistence to antidepressant treatment at month 1, the use of these drugs should be avoided as a rule, given their well-known serious adverse effects.

## Background

While antidepressants have played an important role in the treatment of depression, good adherence to medications is prerequisite to maintain those therapeutic benefits. Despite this self-evident fact, the available evidence has generally shown low adherence rates to antidepressant treatment in patients with a major depressive disorder [1-3]. For example, the adherence rate was found to be 51% at week 16 and further dropped to 21% at week 33 in a sample of 4,312 privately insured outpatients in the US [1]. Similarly, the Vantaa Depression Study showed a one-year continuation rate as low as 50% in Finland (n = 269) [3]. Moreover, Hunot et al. conducted a 6-month prospective study (n = 147) and found that less than half the patients continued use of antidepressant for the 6 months, and only 19% of patients took antidepressants in accordance with clinical guidelines over the study period [4].

It is common to encounter patients who regularly come to see their physicians but do not always take their medications as instructed. To take this fact into consideration, adherence has recently been suggested to be divided into these two components: *persistence *and *compliance *[5]. Persistence is defined as continuously refilling prescriptions in accordance with the suggested duration of the therapy, which is determined by evaluating solely if the patient continues treatment or not [5]. Compliance is the extent in accordance with prescribed dosage and schedule, which can be assessed with Medication Possession Rate (MPR) (i.e., total days a medication was actually dispensed to patients divided by treatment period in days). These two components represent different aspects of patients' behaviours and should be separately assessed, which has been reflected in recent studies in other physical and psychiatric diseases [6,7], including schizophrenia [5] and bipolar disorder [8]. However, surprisingly, there is only one report that has assessed these two components in antidepressant treatment [9]. This study found a MPR of 80% and a 6-month persistence rate of 65.9% in 85 patients with a major depressive disorder who were treated with either fluoxetine or paroxetine in a double-blind, randomized clinical trial. However, these high values would not be expected to thoroughly reflect our daily clinical practice, given that participants in clinical antidepressant trials are more highly motivated and encouraged to receive treatment. Indeed, participants in clinical trials have been suggested to represent a minority of patients in routine clinical practice in terms of psychopathology [10]. In addition, this study did not evaluate effects of clinical characteristics and other antidepressant drugs on persistence and compliance.

To thoroughly evaluate patients' behaviours in taking medications and different profiles in adherence among antidepressants, both persistence and compliance need to be examined in the daily clinical setting. In addition, to further improve our understanding of characteristics of patients' attitudes towards antidepressant treatment, the effects of demographic and clinical characteristics on the persistence and compliance should be investigated, too. For example, a relationship between gender and adherence has not been demonstrated consistently in the literature [11-13]; moreover, any gender-effect specifically on either persistence or compliance has not been investigated. The lack of the data is also true for any aging effect. This paucity of data emphasizes the need of investigations to assess the potential factors associated with good or poor adherence and compliance, which would be expected to provide relevant information for more effective treatment intervention.

To address this gap in the literature, we conducted a systematic chart review and examined persistence and compliance to antidepressant drugs in outpatients with a major depressive disorder in psychiatric clinics in Tokyo, Japan. We also tried to assess the effects of demographic and clinical characteristics on the persistence and compliance.

## Methods

### Patient population

A retrospective chart review was conducted at 3 outpatient clinics: (a) Department of Psychiatry, Saiseikai Central Hospital, Tokyo, Japan, (b) Asakadai Mental Clinic, Saitama, Japan, and (c) Ohizumi Mental Clinic, Tokyo, Japan. Patients who visited one of these participating clinics for the first time between between April 1, 2006 and March 31, 2007 were screened. Among them, outpatients were included if they started to receive antidepressant treatment, were diagnosed with a major depressive disorder (F32 or F33 according to the International Classification of Diseases (ICD), 10^th ^edition), and had not received any psychotropic agents (i.e., antidepressants, mood stabilizers, anxiolytics, and antipsychotics) within the past 6 months before staring antidepressant treatment. Patients who had a past or current history of any concomitant ICD-10 diagnosis or unstable physical conditions that required treatment were excluded. The study was carried out in accordance with the latest version of the Declaration of Helsinki and approved by the institutional review board at all participating sites and exempted from the requirement for informed consent because the study involved de-identified data acquired during routine care.

### Study design

The collected information included age, gender, and psychotropic medications dispensed for 6 months after their first visit. Patients were divided into three age groups for further comparisons, based on their age at the day of their first visit: before age 40, between 40 and 59, and 60 and older, respectively. Daily doses of antidepressants were converted to imipramine equivalents (IMIE) where 100 IMIE mg corresponds to paroxetine 26.7 mg, sertraline 66.7 mg, fluvoxamine 100 mg, milnacipran 100 mg, and sulpiride 200 mg, respectively [14]. Sulpiride has been approved for the treatment of depression as an antidepressant drug in Japan and has been shown to be frequently prescribed [15].

One of the primary outcome measures was the persistence rate to any and initial antidepressant treatments, respectively, 1, 3, and 6 months after their first visit. If patients did not show up for one or more months after their scheduled appointment, it was considered as treatment discontinuation, which is conventional criteria [6,16]. If patients were hospitalized, the date of admission was regarded as that of termination of outpatient treatment. The other primary outcome measure was compliance to any and initial antidepressant treatments, respectively, using a MPR defined as total days a medication was actually dispensed to patients divided by treatment days. For example, when a patient receives a total of 60-day supply of antidepressants until his/her last visit at day 91, the corresponding MPR is calculated to be 0.66 (60/91). Likewise, when a patient receives a total of 160-day supply of antidepressants until his/her last visit at day 183, MPR is 0.87 (160/183). A MPR of 0.8 or more was defined as being compliant to prescribed antidepressant medications [5].

When their initial antidepressant was discontinued or switched to another agent, whether patients had consulted their physicians before quitting the drug or not was recorded based on descriptions of charts. As an initial step, we interpreted qualitative reporting in charts. Participating sites were affiliated with the same academic institution (Keio University, Tokyo, Japan), and physicians working for those participating sites had been educated to recorded reasons of medication change. Where descriptions in charts were insufficient, physicians-of-record were requested to provide additional information.

### Data analysis

Statistical analyses were carried out using the Statistical Package for Social Science (SPSS) version 16.0 for Windows (SPSS Inc., Chicago). Prescribed doses of antidepressants were compared among antidepressants, using an analysis of variance (ANOVA). Logistic regression analysis was also employed to examine predictors of persistence to any antidepressant drug among age group, gender, and the concomitant prescription of benzodiazepine-derivative anxiolytic or hypnotic at a previous survey point. In order to assess predictors of persistence to an initial antidepressant drug, another logistic regression analysis was employed, using those variables described in the previous model as well as an antidepressant drug prescribed on their first visit. A univariate general linear model was used to examine the effects of age group, gender, the use of benzodiazepine-derivative anxiolytic or hypnotic on the MPR of any antidepressant medication. Another univariate general linear model was generated to assess the effects of those variables as well as a first antidepressant drug on the MPR of the initial antidepressant. These general linear models (fixed-effects model) were generated with main effects and all interaction terms. When appropriate, we also examined group differences with pairwise comparisons using the Tukey-Kramer HSD (honestly significant difference). A p-value of < 0.05 was considered statistically significant and all tests were two-tailed.

## Results

### Description of the sample

A total of 2,756 patients that had newly visited one of the participating sites during the targeted period were identified; of these, 1,365 patients were diagnosed with a major depressive disorder. Among them, 998 patients had a prior history of treatment with psychotropic agents within the past 6 months or did not receive antidepressant treatment on the day of their first visit. Charts of the remaining 367 patients were examined. Out of the target sample, the mean ± SD age was 37.6 ± 13.9 years (range, 16 to 82 years), and 46.3% (n = 170) were men. There were no missing data for statistical analyses.

### Initial antidepressant treatment

Sulpiride was the most frequently prescribed antidepressant (40.3%, n = 148), followed by paroxetine (31.1%, n = 114), fluvoxamine (9.3%, n = 34), sertraline (9.0%, n = 33), milnacipran (8.4%, n = 31), amoxapine (1.4%, n = 5), and trazodone (0.5%, n = 2). The mean ± SD starting dose of antidepressants was 54.8 ± 21.1 IMIE mg/day. Significant differences in the initial dose were found among antidepressants (F_(6,360) _= 7.241, p < 0.001); sertraline (40.5 ± 9.9 IMIE mg/day) was lower in relative dose than sulpiride (53.0 ± 23.7 IMIE mg/day, p = 0.03) and paroxetine (62.0 ± 20.0 IMIE mg/day, p = 0.007). Benzodiazepine-derivative anxiolytics and hypnotics were concomitantly prescribed at 33.2% and 48.0%, respectively. There were no differences in benzodiazepine prescription among antidepressants.

### Persistence rates

The ratios of patients who continued an antidepressant treatment are summarized in Tables 1 and 2. The persistence rate to any antidepressant was more than 70% one month after their first visit and gradually declined to slightly less than half at month 6. Two thirds of the patients continued to take their initial antidepressant medication at month 1, and the rate dropped to approximately one third at month 6. Among 252 patients who discontinued their initial antidepressant, 63.1% (n = 159) did so by their own decision without any consultation with their physicians.

**Table 1:** Persistence Rates and Clinical Characteristics

	Persistence Rate (%)		
Baseline	Month 1	Month 3	Month 6

Any antidepressant (n = 367)	72.8	54.0	44.3

Initial antidepressant	66.1	43.3	31.3

Sulpiride (n = 148)	70.9	48.6	35.1

Paroxetine (n = 114)	58.8	36.0	28.1

Fluvoxamine (n = 34)	60.6	29.4	20.6

Sertraline (n = 33)	75.8	51.5	51.5^a^

Milnacipran (n = 31)	67.7	51.6	19.4

Amoxapine (n = 5)	80.0	60.0	20.0

Trazodone (n = 2)	0.0	0.0	0.0

	Persistence Rate to Any Antidepressant (%)		

Sex			

Female (n = 197)	64.4	49.2	40.6

Male (n = 170)	82.3 ^b^	59.4	47.6

Age groups			

Before age 40 (n = 241)	72.6	50.6	41.7

40–59 (n = 95)	75.8	61.1	44.2

60 or older (n = 31)	64.5	58.1	58.1

Anxiolytics use			

No (n = 245)	68.2	51.8	41.6

Yes (n = 122)	82.0 ^c^	58.2	48.4

Hypnotics use			

No (n = 191)	70.2	50.1	40.8

Yes (n = 176)	75.6	49.7	47.2

^a ^Logistic regression analysis found that sertraline use was associated with persistence to an initial antidepressant at month 6 (odds ratio = 2.59 in comparison with sulpiride, 95% CI = 1.16–5.77, p = 0.020).

^b ^Male gender was associated with persistence to any antidepressant drug at month 1 (odds ratio = 2.37, 95% CI = 1.44–3.90, p = 0.001)

^c ^The use of benzodiazepine-derivative anxiolytics also had a positive effect on the persistence to any antidepressant at month 1 (odds ratio = 2.14, 95% CI = 1.22–3.73, p = 0.008).

**Table 2:** Clinical Characteristics Associated with Higher Persistence

	Odds Ratio (95% CI; p value)		
	Persistence to Initial Antidepressant		

	Month 1 ^a^	Month 3 ^b^	Month 6 ^c^

Sulpiride (n = 148)	1.00 (reference)	1.00 (reference)	1.00 (reference)

Paroxetine (n = 114)	0.60 (0.34–1.06; p = 0.08)	0.61 (0.36–1.04; p = 0.07)	0.76 (0.43–1.34; p = 0.35)

Fluvoxamine (n = 34)	0.80 (0.35–1.82; p = 0.59)	0.49 (0.21–1.12; p = 0.09)	0.59 (0.23–1.49; p = 0.27)

Sertraline (n = 33)	1.68 (0.67–4.18; p = 0.27)	1.32 (0.60–2.91; p = 0.49)	2.59 (1.16–5.77; p = 0.02)

Milnacipran (n = 31)	1.13 (0.47–2.71; p = 0.78)	1.40 (0.63–3.13; p = 0.41)	0.56 (0.21–1.49; p = 0.25)

Amoxapine (n = 5)	0.00 (0.0-0.0; p = 0.99)	0.00 (0.0-0.0; p = 0.99)	0.00 (0.0-0.0; p = 0.99)

Trazodone (n = 2)	1.13 (0.12–11.1; p = 0.92)	1.13 (0.18–7.35; p = 0.90)	0.33 (0.03–3.28; p = 0.35)

	Persistence to Any Antidepressant		

	Month 1^d^	Month 3 ^e^	Month 6 ^f^

Sex			

Female (n = 197)	1.00 (reference)	1.00 (reference)	1.00 (reference)

Male (n = 170)	2.37 (1.44–3.90; p = 0.001)	1.42 (0.93–2.17; p = 0.10)	1.29 (0.84–1.97; p = 0.24)

Age groups			

Before age 40 (n = 241)	1.00 (reference)	1.00 (reference)	1.00 (reference)

40–59 (n = 95)	1.01 (0.57–1.79; p = 0.97)	1.43 (0.87–2.33; p = 0.16)	1.02 (0.62–1.66; p = 0.95)

60 or older (n = 31)	0.70 (0.31–1.59; p = 0.40)	1.38 (0.65–2.96; p = 0.41)	1.96 (0.91–4.21; p = 0.08)

Anxiolytics use			

No (n = 245)	1.00 (reference)	1.00 (reference)	1.00 (reference)

Yes (n = 122)	2.14 (1.22–3.73, p = 0.008)	1.30 (0.82–2.04, p = 0.27)	1.38 (0.87–2.17, p = 0.17)

Hypnotics use			

No (n = 191)	1.00 (reference)	1.00 (reference)	1.00 (reference)

Yes (n = 176)	1.44 (0.88–2.36, p = 0.15)	1.24 (0.81–1.90, p = 0.33)	1.35 (0.87–2.17, p = 0.17)

Underlined figures represent statistically significant values.

^a ^Model statistics: χ^2 ^ [11] = 36.0 (p < 0.001); Nagelkerke R^2 ^= 0.13

^b ^Model statistics: χ^2 ^ [11] = 23.8 (p = 0.014); Nagelkerke R^2 ^= 0.084

^c ^Model statistics: χ^2 ^ [11] = 25.8 (p = 0.007); Nagelkerke R^2 ^= 0.095

^d ^Model statistics: χ^2 ^ [5] = 24.3 (p < 0.001); Nagelkerke R^2 ^= 0.093

^e ^Model statistics: χ^2 ^ [5] = 8.4 (p = 0.14); Nagelkerke R^2 ^= 0.03

^f ^Model statistics: χ^2 ^ [5] = 8.0 (p = 0.16); Nagelkerke R^2 ^= 0.03

Odds ratios of significant variables are also summarized in Tables 1 and 2. The use of benzodiazepine-derivative anxiolytics was associated with a higher persistence rate to any antidepressant at month 1, but not thereafter. The use of sertraline had a positive effect on persistence to an initial antidepressant drug at month 6 as compared to sulpiride while other antidepressants failed to show a difference. Males and patients aged ≥ 60 were associated with higher persistence rates to an initial antidepressant drug at month 6 although they failed to show significant effects on 6-month persistence to any antidepressant treatment.

### Medication possession rates

Results of MPRs were summarized in Tables 3 and 4. Although the mean MPR of any antidepressant drug was less than 0.8, 55.6% of patients were considered compliant (i.e., showing a MPR of 0.8 or more). MPRs of the five frequently prescribed drugs were similar to each other without a group difference (Table 3). Univariate general linear models found that gender and age group had significant effects on the MPR of any antidepressant, but failed to find variables that have significant effects on the MPR of an initial antidepressant.

**Table 3:** Medication Possession Rates (MPR) and Clinical Characteristics

	MPR (mean ± SD)
Any antidepressant (n = 367)	0.77 ± 0.28

Initial antidepressant	0.71 ± 0.31

Sulpiride (n = 148)	0.72 ± 0.28

Paroxetine (n = 114)	0.70 ± 0.35

Fluvoxamine(n = 34)	0.64 ± 0.30

Sertraline(n = 33)	0.77 ± 0.28

Milnacipran(n = 31)	0.78 ± 0.30

Amoxapine (n = 5)	0.64 ± 0.27

Trazodone (n = 2)	0.68 ± 0.64

	MPR of any antidepressant

Sex ^a^	

Female (n = 197)	0.73 ± 0.29

Male (n = 170)	0.81 ± 0.26

Age group ^b^	

Before age 40 (n = 241)	0.76 ± 0.28

40–59 (n = 95)	0.76 ± 0.25

60 or older(n = 31)	0.87 ± 0.29

Anxiolytics use	

No (n = 245)	0.77 ± 0.29

Yes (n = 122)	0.77 ± 0.25

Hypnotics use	

No (n = 191)	0.78 ± 0.29

Yes (n = 176)	0.76 ± 0.27

^a^Univariate general linear model found that gender had significant effects on the MPR (F_(1,343) _= 4.43; Corrected Model: F_(23,343) _= 1.78, p = 0.016, R^2 ^= 0.11).

^b ^Univariate general linear model found that age group had significant effects on the MPR (F_(2,343) _= 3.39, p = 0.035; Corrected Model: F_(23,343) _= 1.78, p = 0.016, R^2 ^= 0.11).

**Table 4:** Effects of Clinical Characteristics on Medication Possession Rates (MPR)

	F value	P value
	Effects on MPR of initial antidepressant^a^	

Antidepressant	F_(6,279) _= 0.87	0.52

Sex	F_(1,279) _= 0.79	0.38

Age group	F_(2,279) _= 3.36	0.036 ^b^

Anxiolytics use	F_(1,279) _= 0.88	0.35

Hypnotics use	F_(1,279) _= 0.08	0.77
	Effects on MPR of any antidepressant^c^	

Sex	F_(1,343)_ = 4.43	0.036

Age group	F_(2,343)_ = 3.39	0.035

Anxiolytics use	F_(1,343) _= 1.24	0.27

Hypnotics use	F_(1,343) _= 0.075	0.78

Underlined figures represent statistically significant values.

^a^Univariate general linear model, Corrected Model: F_(87,279) _= 1.29, p = 0.07, R^2 ^= 0.29

^b^Although the p-value was < 0.05, the model statistics failed to show any statistical significance as described above.

^c^Univariate general linear model, Corrected Model: F_(23,343) _= 1.78, p = 0.016, R^2 ^= 0.11

## Discussion

We conducted the first study that investigates persistence and compliance to antidepressants in patients with a major depressive disorder in the clinical outpatient setting. Notwithstanding the limitations imposed by the retrospective design as discussed below, the finding of low persistence and insufficient compliance to antidepressants in this study suggests that patients with depression should be encouraged more to derive the full benefits from their antidepressant treatment. In addition, males were associated with a higher persistence and better compliance rate to antidepressant treatment. A significant association was also found between the use of benzodiazepine-derivative anxiolytics and a higher persistence rate in the beginning of the treatment. While a causal attribution cannot be made in light of the retrospective nature of this study, the results highlight the critical importance of devising effective treatment strategies to enhance patients' adherence to medications in patients with depression.

The overall persistence rate at month 6 was found to be as low as 44% in the present study, which is comparable to 42–51% reported in previous surveys that were conducted in clinical settings [1-3]. Also, persistence rates to initial antidepressants were similar to those in the literature (20–40%) [17,18]. Besides these low persistence rates, another concern is an insufficient compliance to antidepressants, as slightly more than half the patients were considered compliant. Poor compliance has been a major obstacle, especially in the treatment of chronic disease; indeed, low rates of compliant patients have been reported in chronic physical illnesses, including hypertension (50–65%) [6,7] and osteoporosis (53%) [16]. Given that major depressive disorders frequently need a long-term antidepressant treatment for relapse prevention [19,20], these illnesses should be regarded as another chronic condition. Notwithstanding this, the rate of compliant patients in this study (56%) is lower than in schizophrenia (79%) [5] and epilepsy (74%) [21] for which compliance to the medication has been more seriously considered and targeted. These findings point to a necessity of targeting the medication adherence in depression more thoroughly than we currently do.

Another important finding is more than 60% of dropouts discontinued their initial antidepressant treatment without consulting with their physicians. This high rate may suggest insufficient communication between patients and their physicians. One interesting survey, including telephone interviews in 401 patients with depression and written surveys in 137 prescribing physicians, showed that 72% of physicians reported that they usually asked patients to continue using antidepressants for at least 6 months, but only 34% of patients reported that their physicians educated them to continue using antidepressants for this duration and 56% reported receiving no instructions [22]. Further, patients who said they were told to take their medication for less than 6 months were 3 times more likely to discontinue therapy, as compared with patients who said they were told to continue therapy longer. The percentage of 60% that we found in our study could have been reduced, to some extent, with appropriate instructions to patients. Elwyn et al. suggested that the involvement of patients in shared decision making is one of the key components of enhancing adherence [23]. Combined with the findings of those previous studies [22,24], our results emphasize the need of a much closer liaison between patients and their physicians.

A positive effect of the use of benzodiazepine-derivative anxiolytics on the persistence to antidepressant treatment was found at month 1. Although benzodiazepines themselves have little or no antidepressant effect, their anxiolytic and hypnotic effects could diminish associated symptoms such as anxiety and insomnia before antidepressants start to exert their effects, which in turn may result in better persistence to the treatment. In fact, Furukawa et al. reported that patients treated with combination therapy with a benzodiazepine were 37% less likely to dropout than with an antidepressant alone [25]. However, the benefits of benzodiazepines seem to be lost beyond one month as no significant effect was observed at months 3 and 6. Physicians should also keep in mind that adverse effects of benzodiazepines are potentially very serious. Benzodiazepines have been reported to impair cognitive function, including memory [26], and to be associated with dependence [27]. Further, they increase the risk of falls [28], which may well be associated with a high mortality rate in the elderly. Considering an absolute difference in the 1-month persistence rate as low as 14 points in percentage between benzodiazepine users and non-users, the benefit of using this drug may be limited. Given those serious side effects and limited potential benefits, the use of benzodiazepines should not be justified as a rule.

We also found that the use of sertraline might predict a long-term persistence to antidepressant treatment. Given that persistence rates or time to discontinuation could be utilized as a relevant outcome measure as recent large-scaled clinical trials do [29,30], sertraline may be a good first-line antidepressant drug in the treatment of depression. Poorer efficacy and a higher frequency of side effects are known to be linked to a lower persistence rate in the treatment with antidepressants [31]. A reported better side effect profile in sertraline [11] may contribute to our finding. However, these preliminary results should be interpreted with caution since we did not directly evaluate therapeutic and adverse effects of these medications in this study. In fact, the finding of varying persistence rates across medications could reflect confounding by the provider effect, and sertraline is the newest antidepressant that became available in 2006 in Japan. We cannot deny a possibility that physicians might have titrated the dose of this newer drug more carefully than the other older drugs, resulting in the higher persistence to sertaline that we observed. The fact that the initial dose of sertraline was lower than that of sulpiride may support this notion. In addition, although patients who had received psychotropic drugs within the past 6 months were excluded from this survey, we cannot reject a possibility that some of the patients had received prior antidepressant treatment in their previous episodes, if any, which might have affected the outcomes. Cost of medications is also expected to affect persistence to antidepressant treatment in many clinical settings. However, this is unlikely the case in the present study since at least 70% of medication fees are covered by the national insurance system in Japan.

Males showed a higher persistence and better compliance in this study; however, this relationship has not been consistent in the literature [11-13]. Burra et al. reported that, consistent with our study, male patients were more compliant to prescribed medications than female patients in 80 patients in Canada [11]. On the other hand, male gender was reported to be associated with a greater likelihood of discontinuation in the US (n = 406) [12] and Belgium (n = 66) [13]. These discrepant findings may be derived from different social and cultural backgrounds. We also found a higher persistence rate and MPR in older patients, which is in line with more positive attitudes towards antidepressants in this population [3]. Older people's literally compliant behaviours may explain this finding. However, these potential causes of this gender difference in persistence and compliance remain speculative and warrant further investigations.

There are several limitations to be noted in this study. First, the results in the present study should be interpreted in light of the relatively small sample size. Second, information on many clinical and demographic characteristics was lacking. Moreover, as we used broad exclusion criteria such as a past or current history of any concomitant ICD-10 diagnosis or unstable physical conditions, the sample collected in this study might not have been representative of the entire general population with depression. Furthermore, behaviours towards treatment are expected to be subject to patients' social and cultural backgrounds. These limitations indicate a necessity of further information from various clinical settings in order to provide a robust agreement on this issue. Third, patients were not randomized to the antidepressants, and physicians' prescribing behaviours may reflect a bias in training and both direct and indirect influence of current and local standard of care [32]. Since different treatment sites may have an impact on patients' behaviours towards antidepressant treatment, we performed additional analyses, including site as a parameter. Although we did not find any significant effect of this factor on persistence rate or MPR, the relationship between patients and their physicians is still likely to affect treatment discontinuation. Fourth, treatment discontinuation was defined as not showing up for one or more months after their scheduled appointment. Although this definition has conventionally been used in this type of study [6,16], some patients might have continued treatment at another facility. Furthermore, refill patterns do not directly measure compliance or even persistence with certainty. Fifth, the retrospective nature of this survey did not allow us to evaluate the effectiveness of drug treatment, and treatment intervention was not standardized. Most importantly, we do not have data on actual clinical outcomes. Some of the patients who were regarded as dropouts in the present study might have discontinued their treatment because of an improvement in their illness although antidepressant treatment should be continued for more than 6 months to prevent a relapse [19,20,33]. Further, patients might not have taken their medications even though they showed up for appointments. However, these limitations are not unique to our study. Indeed, the key studies assessed patients' adherence to medications retrospectively [5-7,21]. Despite this positive indication, our preliminary findings need to be replicated in a larger number of samples, using a prospective study design. Finally, an association between persistence and compliance was not evaluated since these two outcome measures were influenced by the same factor, sex. Further invistigations are warranted to evaluate the interaction between these two measures, which will provide a better understanding of patients' behaviours towards antidepressant treatment.

## Conclusion

We examined persistence rates and MPRs in 367 patients with a major depressive disorder. Low persistence and poor compliance to antidepressant treatment were found to be problematic in patients with depression in the clinical outpatient setting. Porer compliance to medications in patients with depression than in those with schizophrenia or epilepsy points to a necessity of targeting the medication compliance in this chronic illness, too. Since there is a high rate of antidepressant discontinuation without any consultation with physicians, closer communication between patients and their physicians should be encouraged, which in turn would be expected to enhance persistence to medications and hence improve outcomes. The use of sertraline may also enhance persistence to antidepressant treatment. Future prospective studies are warranted to further address these preliminary findings.

## Competing interests

NS has received speaker's honoraria from Pfizer and received manuscript fees from Dainippon Sumitomo Pharma within the past 5 years. HU's fellowship has been supported by the Japanese Society of Clinical Psychopharmacology, the Pfizer Health Research Foundation, and the Mochida Memorial Foundation. HU has received manuscript fees from Otsuka and Dainippon Sumitomo Pharma within the past 5 years. KW has received grants, consultant fees from Janssen Pharma, Eli Lilly, Pfizer, GlaxoSmithKline, and Dainippon Sumitomo Pharma, and received speaker's honoraria from Janssen Pharma, Eli Lilly, Meiji, Astellas Pharma, Yoshitomi, Dainippon Sumitomo Pharma, Otsuka, Pfizer, and GlaxoSmithKlein within the past 5 years. TK has received a research grant from GlaxoSmithKlein within the past 5 years. TS, TH, and HK have nothing to disclose.

## Authors' contributions

NS, KW, and TK contributed to and have approved the design and the protocol of the study. NS, HU, and TS contributed to the literature searches and analyses. NS wrote the first draft of the manuscript, and all authors contributed to and have approved the final manuscript.

## Pre-publication history

The pre-publication history for this paper can be accessed here:


